# “Functional Fitness Training”, CrossFit, HIMT, or HIFT: What Is the Preferable Terminology?

**DOI:** 10.3389/fspor.2022.882195

**Published:** 2022-05-26

**Authors:** Fábio Hech Dominski, Ramires Alsamir Tibana, Alexandro Andrade

**Affiliations:** ^1^Laboratory of Sport and Exercise Psychology, Human Movement Sciences Graduate Program, College of Health and Sport Science, Santa Catarina State University (UDESC), Florianópolis, Brazil; ^2^Physical Education Department, Univille University, Joinville, Brazil; ^3^Graduate Program in Health Sciences, Faculty of Medicine, Federal University of Mato Grosso (UFMT), Cuiabá, Brazil

**Keywords:** physical activity, high-intensity functional training, exercise, physical fitness, strength training

## Introduction

Our objective in this opinion is initially to analyze the terminology related to one of the main trends in exercise science and practice and then to propose a term that could be deemed preferable considering the comprehensive approach of this type of training.

Recently, from the 2000's onwards, a new exercise trend emerged worldwide, driven mainly by a brand, to improve physical fitness through the optimization of several components, such as aerobic capacity, muscular strength and endurance, speed, coordination, agility, balance, flexibility, and stamina (Glassman, 2002). This trend revolutionized the fitness world. Until its inception, no type of training had included so many components of physical fitness in the same training session, with participation of populations with different fitness levels. To perform this training, sessions include a wide range of functional movements involving the whole body and universal motor recruitment patterns, including some activities that can be extrapolated to daily life. There are activities such as calisthenics, strength/power, weightlifting, gymnastic movements, plyometric exercises, cycling, running, and rowing, which can be performed at a high intensity (Tibana et al., 2019). Challenge, scalability, enjoyment, affiliation, and the constant variation of workouts are characteristics that may explain the exponential growth among practitioners of different levels of physical fitness (Dominski et al., 2020), including a wide range of populations, including healthy individuals, obese individuals, and athletes. The growth in training with high intensity has aroused the interest of researchers, including our research group, mainly focusing on psychological (Dominski et al., 2020) physiological benefits (Tibana et al., 2022) as well as injuries (Dominski et al., 2021).

Several terms to denominate this type of training have been used both in science and practice. The terminology (terms) is provided in Table 1. In practice, the term CrossFit^®^, which is a company, it is widely used in situations covering from the media to informal conversations, as well as in scientific research. However, in recent years, in research and practice, we have seen an increase in the variety of terms used, sometimes to describe the same thing, but also to describe different types of fitness training programs, including CrossFit^®^, high-intensity functional training (HIFT), high-intensity multimodal training (HIMT), functional fitness training (FFT), extreme conditioning program (ECP), and Mixed Modal Training.

**Table 1:** Terminology.

**Terms**	**Definition**	**Organization/reference**
CrossFit^®^	CrossFit^®^ is a strength and conditioning system built on constantly varied, if not randomized, functional movements executed at high intensity.	CrossFit^®^ Inc. Glassman, 2007
High-intensity multimodal training (HIMT)	HIMT involves exercise programs that mix many different exercise modalities (e.g., weightlifting, powerlifting, gymnastic, calisthenics, plyometric, running, and others) and train multiple physical capacities at the same time (e.g., cardiorespiratory, muscle strength, and flexibility) HIMT encompasses all relevant styles of combined aerobic, resistance and/ or bodyweight training (i.e., HIFT, bodyweight HIIT, CrossFit^®^) performed at a high or vigorous intensity	Carnes and Mahoney, 2019 Sharp et al., 2022
Extreme conditioning programs (ECP)	High-volume aggressive training workouts that use a variety of high-intensity exercises and often time a maximal number of repetitions with short rest periods between sets.	Bergeron et al., 2011
Functional fitness	A sport that aims to develop athletes' proficiency across a variety of movement patterns, activities, and energy systems. Training must develop the competency in various realms, including demonstrations of their aerobic capacity, strength, bodyweight endurance, bodyweight skill, mixed modal capacity, and power.	The International Functional Fitness Federation, iF3
High-intensity functional training (HIFT)	A training style [or program] that incorporates a variety of functional movements, performed at high intensity [relative to an individual's ability], and designed to improve parameters of general physical fitness (e.g., cardiovascular endurance, strength, body composition, flexibility, etc.) and performance (e.g., agility, speed, power, strength, etc.)	Feito et al., 2018
Mixed modal training	An approach that combines several physical training modalities in a single program	Marchini et al., 2019

Despite this, according to Sharp et al. (2022), there is a lack of an operational term that broadly encompasses all types of exercise and physical fitness. Therefore, there is no full agreement among the scientists and athletes or the community (Schlegel, 2020). Recently, different definitions have emerged in some articles published, and this letter to the editor proposes a discussion of these terms, in addition to a proposition of a preferable term.

## Discussion

**Figure 1:**
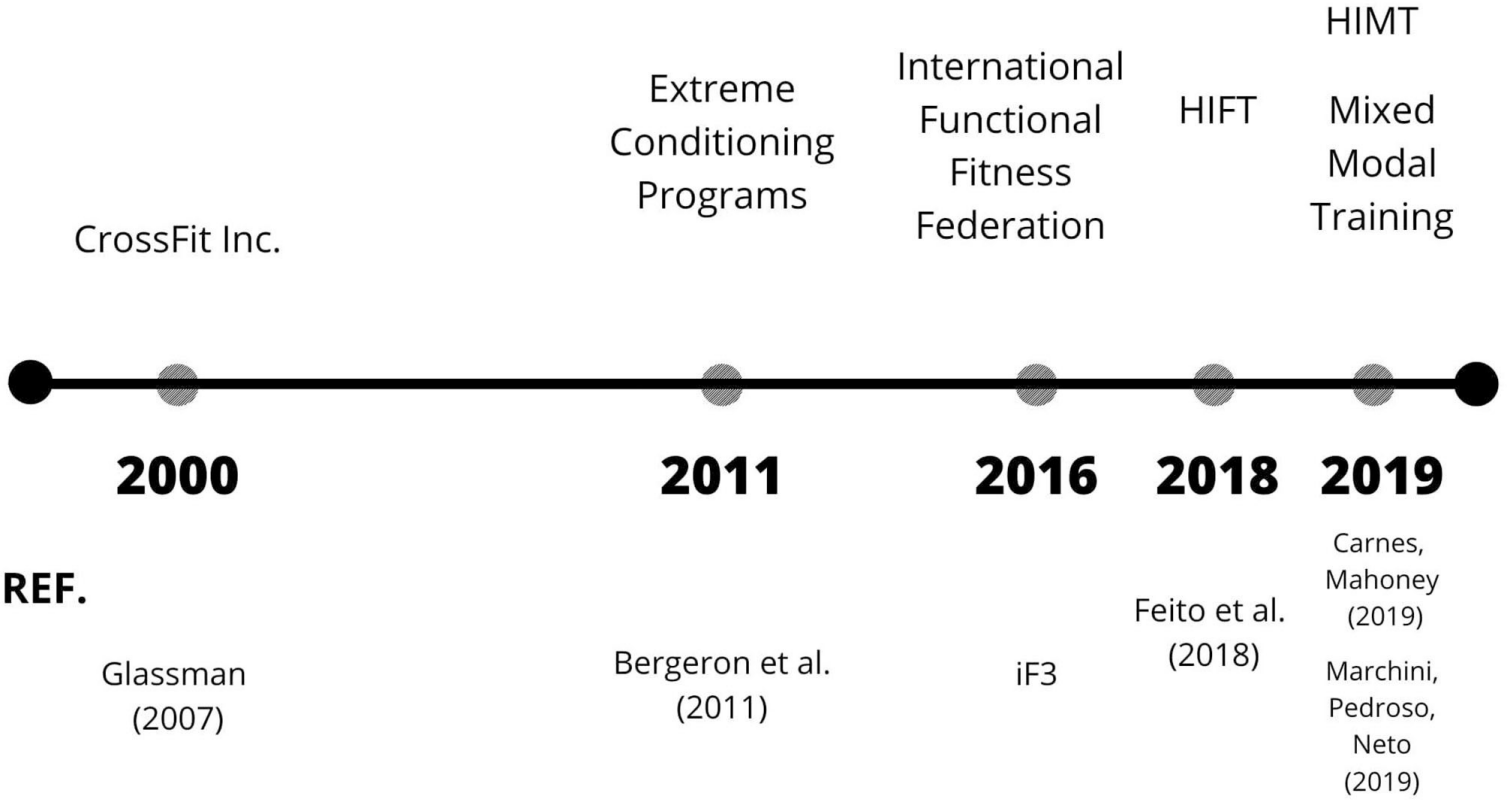
Timeline of appearance of terms and references.

In a quick search in the PubMed database to gain an overview of the number of studies (title field on 14th April, 2022), we observed different results according to the terms searched. We chose the terms in accordance with those discussed here. The search “crossfit” resulted in 101 documents, while “functional fitness” resulted in 75, “functional fitness training” in 2, “high-intensity functional training” in 29, “extreme conditioning program^*^” in 6, “high-intensity multimodal training” and “Mixed Modal Training” showed no results. Below we will discuss some aspects of each of the terms, thus since 2000 additional terms have been used over time, as shown in Figure 1 (Timeline of appearance of terms and the respective references).

CrossFit^®^ is a company (CrossFit^®^ LLC) founded in 2000, but its inception by Greg Glassman occurred in 1996 in the USA. According to Official CrossFit^®^ Affiliate Map (2022) there are more than 11,000 affiliated gyms worldwide (Official CrossFit Affiliate Map, 2022). CrossFit^®^ is composed of a central branded organization (CrossFit^®^ Headquarters) and a network of licensed affiliates–the gyms that are unofficially called boxes (Edmonds, 2021). Regarding modality, CrossFit^®^ has a hybrid nature as both sport and exercise, due to the mixture of elite sport and practice for the health, enjoyment and conditioning of the general population (Edmonds, 2021). The key aspects of CrossFit^®^ are constantly varied, high intensity, functional movement, but according to Edmonds (2021) (Edmonds, 2021) simply performing training with these characteristics does not mean one is doing CrossFit^®^ - CrossFit Inc. needs to legally recognize as an official affiliate. To use the term CrossFit^®^ some aspects should be respected: the affiliated gym, the CrossFit^®^ methodology designed by the brand (official classes and/or exercises), and the inclusion of a certified CrossFit trainer. The affiliation of a physical location used for training allows the owners to legally use the CrossFit^®^ trademark subject to the fulfillment of several requirements, such as obtaining at least a Level 1 Certificate to teach, some personal factors (such as the background, what CrossFit affiliation means to the coach, among others), the cost of affiliation through a fee payment, as well as criteria related to location and insurance. If an individual or entity is using CrossFit's intellectual property (e.g., trademarks or copyrights) without a license, confidential reports can be submitted to the legal department.

Although widely used, the brand name CrossFit^®^ may decrease over time in publications, mainly due to the researchers' fear of possible lawsuits from CrossFit^®^ Inc., but also from publishers and scientific journals (Tibana et al., 2016), which is becoming increasingly common. CrossFit^®^ is a term that, unlike others, does not come from research but exclusively from the brand. Some journals, in the evaluation process, ask for confirmation that the study incorporates and/or assesses the true nature of the CrossFit LCC brand, including the gym where the exercise regime took place, official classes and/or exercises, and a certified CrossFit trainer. In this sense, we (Tibana et al., 2016) were instructed to change the title of our article (from CrossFit to extreme conditioning programs) because the data collection was not performed in a CrossFit^®^ affiliated box from CrossFit^®^ LLC. According to the guidelines given by the brand itself at the time of this publication, a workout can only be described as “CrossFit” if it is executed by CrossFit Inc., or by a group licensed by CrossFit Inc., including the journal's guidance to use the term “fitness training.” Thus, the scientific community has adopted other terms, either under the guidance of the journal editors and reviewers and/or because the authors decided to definitely use another term to describe this type of training.

In 2011 a group of researchers published a Consortium for Health and Military Performance and American College of Sports Medicine consensus paper on extreme conditioning programs in military personnel (Bergeron et al., 2011). The term extreme conditioning program, as discussed previously by Feito and Mangine (2017), is certainly misleading and might be misinterpreted. Although a few authors have referred to CrossFit^®^ as being extreme, it may be more appropriately referred to as “metabolic conditioning,” “high-intensity functional training,” or as its military roots have termed, “general physical preparedness” (Glassman, 2002). Here, the term metabolic conditioning is used in a context-specific and practice-oriented sense, reflecting its widespread and conventional usage in functional fitness environments. We infer that the term “extreme” automatically suggests abnormal activity, when in reality this is a training methodology currently employed by hundreds of thousands of individuals worldwide, with different levels of physical fitness, with scalability (modification options for the activities, movements, and exercises) being a strong point of the methodology.

Later, other groups of researchers adopted terms referring to high intensity, such as: HIFT (Feito et al., 2018) and HIMT (Carnes and Mahoney, 2019; Gentil et al., 2021). Recently, Carnes and Mahoney (2019); Gentil et al. (2021) and Sharp et al. (2022) used HIMT and included in this definition several terms such as CrossFit^®^, HIFT, bodyweight HIIT, cross-training, and others. Feito et al. (2018) proposed a definition to guide future publications about this training style [or program] as HIFT: “HIFT incorporates a variety of functional movements, performed at high-intensity [relative to an individual's ability], and designed to improve parameters of general physical fitness (e.g., cardiovascular endurance, strength, body composition, flexibility, etc.) and performance (e.g., agility, speed, power, strength, etc.).” However, these terms are associated only with a specific part of the modality's training session, popularly known as metabolic conditioning (METCON), as recognized recently by Sharp et al. (2022). Usually this part corresponds to the end of the training session in the gyms (CrossFit^®^ affiliated or not). Thus, the use of the terms HIFT and HIMT would be correct only when the authors referred to METCON performed at high-intensity and not the modality in a broad way, as it includes the development of power, strength, and cardiovascular fitness in both periodization and training sessions.

Recently, Ide et al. (2022), recommended that the terms functional training, high-intensity functional training, and functional fitness training no longer describe any physical training program. The exercise programs, according to the authors, can be classified as strength, power, endurance, and flexibility. However, the authors do not suggest any specific term to describe a comprehensive type of training which includes strength, power, endurance, and metabolic conditioning training. However, mentioning specific types of exercises performed, as suggested by Ide et al. (2021), such as strength, plus endurance, or others, it is not viable. Furthermore, contrary to what Ide et al. (2022) claim, functional fitness training is not HIFT, and the difference found between functional fitness training and HIFT (the specific part of the modality's training session known as METCON) programs is consistent, as described above. Furthermore, Ide et al. (2022) affirmed that functional fitness training “could be easily described as strength training.” However, the development of strength is only a specific part of the training session, as shown in Table 2 (e.g., bench press and front squat).

**Table 2:** Functional fitness training in a week (designed for one person).

	**Monday**	**Tuesday**	**Wednesday**	**Thursday**	**Friday**	**Saturday**	**Sunday**
Weightlifting	Snatch High Pull + Hang Snatch High Pull + Hang Snatch + Snatch 50%-1 55%-1 60%-1 65%-1 65%-1 70%-1	High Hang Clean + Hang Clean + Clean + Split Jerk 50%-1 55%-1 60%-1 65%-1 65%-1 70%-1	Accessory Exercises EMOM 18 min, rotating: 1) 10 GHD Hip Extension 2) 10 Mini Band Wall Slides 3) 10 Banded Face Pulls 4) 10 Drop and catch in 90° of shoulder abduction (R/L) 5) 15m Dumbbell Overhead Carry 6) Rest	Rest	Power Clean + Split Jerk 50%-3 (2x) 55%-3 (2x) 60%-3 (2x) 65%-3 (2x)	Accessory Exercises EMOM 18 Min, rotating: 1) 5 Single Arm Front Rack Curtsy Lunge 2) 30 m Dual KB Front Rack Carry 3) 10 Psoas March (each side) 4) 30” Glute Bridges Hold 5) 10 Rower Pike Up 6)-Rest	Rest
Strength	Bench Press 50%-3 60%-3 70%-3 80%-3 85%-2	Front squat 50%-3 60%-3 70%-3 80%-3 85%-2			RDL (%1RM Front Squat) 50%-3 60%-3 70%-3 80%-3 85%-3		
Gymnastic conditioning	4 Sets: 1 Bar Muscle-Up 2 Toes to Bar 3 Chest to Bar Pull-ups 2 Toes to Bar 1 Bar Muscle-Up Rest as little as needed between unbroken sets.		-		AMRAP 5 min 21 Burpees 21 Pull ups 21 Double Dumbbell Deadlift Rest 2 Min AMRAP 4 min: 15 Burpees 15 Pull ups 15 Double Dumbbell Deadlift Rest 2 Min AMRAP 3 min: 9 Burpees 9 Pull ups 9 Double Dumbbell Deadlift	Reckless 50 Wall Ball (9/6 kg) 40 Cal Row 30 Dual Dumbbell Box Step Up (22/16 kg) 30 meters Hand Stand Walking (5 meters segment unbroken) 30 Dual Dumbbell Box Step Up (22/16 kg) 40 Cal Row 50 Wall Ball	
Metabolic conditioning	3 Rounds: 20 Toes to bar 50 Double unders	“Death by Triplet” Complete as many rounds as possible during 20 min .8 Burpee Box Jump Overs/ 8 Hang Power Cleans (40/30 kg) / 8 Thrusters (40/30 kg)					
Aerobic conditioning	BikeErg Workout 2 x 5,000 m	Rowing Workout 2 x 3,000 m	Running Workout 4,000 m -		SkiErg Workout 20 min		

*AMRAP, As many repetitions as possible; EMOM, Every Minute on The Minute; GHD, Glute Ham Developer; KB, Kettlebell; L, Left; R, Right; RDL, Romanian deadlift*.

We propose the adoption of “functional fitness training (FFT)” as the preferable term, at the present to describe this comprehensive type of training, characterized by a variety of movement patterns (see some examples in Table 2 – which is illustrative rather than prescriptive, with the aim to illustrate the scope and diversity of training components encompassed by the proposed terminology), activities (which include weightlifting, strength, gymnastic conditioning, metabolic conditioning, aerobic conditioning), and energy systems used (ATP-CP/phosphagen, glycolytic, and oxidative). This term is based on two other terms: functional training and physical fitness. The distinction of aerobic conditioning and metabolic conditioning is not based on bioenergetic exclusivity, but rather on training structure, intensity distribution, and dominant energetic contribution. Aerobic conditioning refers to continuous or interval-based efforts designed to maximize oxidative capacity over longer durations, while metabolic conditioning refers to mixed-modal, high-intensity bouts where aerobic metabolism contributes substantially but is not the sole or dominant limiter.

Functional training can be understood, although with different definitions, as a way to increase performance in some functional tasks (e.g., activities of daily living or tests related to athletic performance) (Fleck and Kraemer, 2014). This definition was pointed out as the most rational definition of functional training according to Ide et al. (2022). Part of this term is due to the concept of physical fitness. According to Caspersen et al. (1985) physical fitness is a set of attributes related to health (such as cardiorespiratory endurance, muscular endurance, muscular strength, body composition, and flexibility) or skills (athletic ability), both present in functional fitness training, even when considering only one training session.

Functional fitness training is the most comprehensive and inclusive term to describe the variety of activities performed (see an example of training in Table 2). Functional fitness training must develop the people's competency in various realms, including demonstrations of aerobic capacity, strength, bodyweight endurance, bodyweight skills, and power. In this sense, CrossFit^®^ is a type of functional fitness training.

Furthermore, there is an International Functional Fitness Federation, the iF3, which is the International Governing Body for Competitive Functional Fitness (The International Functional Fitness Federation, iF3). A specific organization can provide support to fuel the growth of functional fitness as a sport. This is an organization which aims to implement a standardized rulebook and clear movement standards. In addition, this organization has written safety guidelines for event organizers and increased competitive opportunities for athletes, being composed of several committees (technical, adaptive, medical, gender equality, athletes, and ethics–including a set of Anti-Doping Rules). There are several current national federations recognized by the International Federation (4 in Africa, 14 in America, 8 in Asia, 25 in Europe, and 1 in Oceania), totalizing 52 countries.

Functional fitness training was also recognized and regarded as one of the Top 20 Worldwide Fitness Trends for 2022. This trend first appeared in the ranking in 2007 and currently appears as trend n.14 (Thompson Walter, 2007). The limitation in the use of the proposed term is temporal. It may take time to establish the term compared to the brand.

It is relevant to agree on a new term to describe this type of training both in research and practice, considering sports scientists who are investigating this type of training as a “sport,” and practitioners, athletes, and spectators interested in the practice, so they know what it is just by the term. The standardization of a term helps in research, because when we adopt only one term, it is possible to promote consistency in study protocols, to aid comparisons, and to find a greater number of articles in a search in databases, both for original articles and for the writing of systematic reviews. Regarding practitioners, the adoption of a term like the one proposed here, avoids that this type of training is linked to a brand, as it has been since then, which is susceptible to different interests–administrative and management, political, financial and others.

Considering the analysis about the terminology related to one of the main trends in exercise science and practice, we propose that the term functional fitness training could be more suitable than CrossFit^®^, HIFT, HIMT, or others.

## Author Contributions

FD: writing (review and editing) of the preliminary text and of the article. RT: idea conception and writing (review and editing). AA: supervision. All authors made significant individual contributions to this manuscript.

## Funding

This research was funding by the Coordenação de Aperfeiçoamento de Pessoal de Nível Superior—Brazil (CAPES) and FAPESC—Foundation for research and innovation support of the State of Santa Catarina—Grant Number 2019031000035 and call number N° 027/2020.

## Conflict of Interest

The authors declare that the research was conducted in the absence of any commercial or financial relationships that could be construed as a potential conflict of interest.

## Publisher's Note

All claims expressed in this article are solely those of the authors and do not necessarily represent those of their affiliated organizations, or those of the publisher, the editors and the reviewers. Any product that may be evaluated in this article, or claim that may be made by its manufacturer, is not guaranteed or endorsed by the publisher.

## Correction note

A correction has been made to this article. Details can be found at: 10.3389/fspor.2026.1888508.
