# Correction: “Functional fitness training”, CrossFit, HIMT, or HIFT: what is the preferable terminology?

**DOI:** 10.3389/fspor.2026.1888508

**Published:** 2026-06-12

**Authors:** Fábio Hech Dominski, Ramires Alsamir Tibana, Alexandro Andrade

**Affiliations:** 1 Laboratory of Sport and Exercise Psychology, Human Movement Sciences Graduate Program, College of Health and Sport Science, Santa Catarina State University (UDESC), Florianópolis, Brazil; 2 Physical Education Department, Univille University, Joinville, Brazil; 3 Graduate Program in Health Sciences, Faculty of Medicine, Federal University of Mato Grosso (UFMT), Cuiabá, Brazil

**Keywords:** physical activity, high-intensity functional training, exercise, physical fitness, strength training

A correction has been made to the section **Introduction**, Paragraph 1:

“Our objective in this opinion is initially to analyze the terminology related to one of the main trends in exercise science and practice and then to propose a term that could be deemed preferable considering the comprehensive approach of this type of training.”

A correction has been made to the section **Discussion**, Paragraph 4:

“In 2011 a group of researchers published a Consortium for Health and Military Performance and American College of Sports Medicine consensus paper on extreme conditioning programs in military personnel (Bergeron et al., 2011). The term extreme conditioning program, as discussed previously by Feito and Mangine (2017), is certainly misleading and might be misinterpreted. Although a few authors have referred to CrossFit® as being extreme, it may be more appropriately referred to as “metabolic conditioning,” “high-intensity functional training,” or as its military roots have termed, “general physical preparedness” (Glassman, 2002). Here, the term metabolic conditioning is used in a context-specific and practice-oriented sense, reflecting its widespread and conventional usage in functional fitness environments. We infer that the term “extreme” automatically suggests abnormal activity, when in reality this is a training methodology currently employed by hundreds of thousands of individuals worldwide, with different levels of physical fitness, with scalability (modification options for the activities, movements, and exercises) being a strong point of the methodology.”

A correction has been made to the section **Discussion**, Paragraph 7:

“We propose the adoption of “functional fitness training (FFT)” as the preferable term, at the present to describe this comprehensive type of training, characterized by a variety of movement patterns (see some examples in Table 2 – which is illustrative rather than prescriptive, with the aim to illustrate the scope and diversity of training components encompassed by the proposed terminology), activities (which include weightlifting, strength, gymnastic conditioning, metabolic conditioning, aerobic conditioning), and energy systems used (ATP-CP/phosphagen, glycolytic, and oxidative). This term is based on two other terms: functional training and physical fitness. The distinction of aerobic conditioning and metabolic conditioning is not based on bioenergetic exclusivity, but rather on training structure, intensity distribution, and dominant energetic contribution. Aerobic conditioning refers to continuous or interval-based efforts designed to maximize oxidative capacity over longer durations, while metabolic conditioning refers to mixed-modal, high-intensity bouts where aerobic metabolism contributes substantially but is not the sole or dominant limiter.”

The original version of this article has been updated.

